# Quality improvement process to assess tattoo alignment, set‐up accuracy and isocentre reproducibility in pelvic radiotherapy patients

**DOI:** 10.1002/jmrs.79

**Published:** 2014-11-21

**Authors:** Kelly Elsner, Kate Francis, George Hruby, Stephanie Roderick

**Affiliations:** ^1^ Sydney Cancer CentreDepartment of Radiation OncologyRoyal Prince Alfred HospitalSydneyAustralia; ^2^ Radiation OncologyThe Canberra HospitalGarranAustralia; ^3^ University of SydneySydneyAustralia; ^4^ Northern Sydney Cancer CentreRoyal North Shore HospitalSt LeonardsAustralia

**Keywords:** Isocentre definition, pitch, roll, yaw

## Abstract

**Introduction:**

This quality improvement study tested three methods of tattoo alignment and isocentre definition to investigate if aligning lateral tattoos to minimise pitch, roll and yaw decreased set‐up error, and if defining the isocentre using the lateral tattoos for cranio‐caudal (CC) position improved isocentre reproducibility. The study population was patients receiving curative external beam radiotherapy (EBRT) for prostate cancer. The results are applicable to all supine pelvic EBRT patients.

**Methods:**

The three sequential cohorts recruited 11, 11 and 10 patients respectively. A data set of 20 orthogonal pairs of electronic portal images (EPI) was acquired for each patient. EPIs were matched offline to digitally reconstructed radiographs. In cohort 1, lateral tattoos were adjusted to minimise roll. The anterior tattoo was used to define the isocentre. In cohort 2, lateral tattoos were aligned to minimise roll and yaw. Isocentre was defined as per cohort 1. In cohort 3, lateral tattoos were aligned as per cohort 2 and the anterior tattoo was adjusted to minimise pitch. Isocentre was defined by the lateral tattoos for CC position and the anterior tattoo for the left–right position.

**Results:**

Cohort 3 results were superior as CC systematic and random set‐up errors reduced from −1.3 mm to −0.5 mm, and 3.1 mm to 1.4 mm respectively, from cohort 1 to cohort 3. Isocentre reproducibility also improved from 86.7% to 92.1% of treatment isocentres within 5 mm of the planned isocentre.

**Conclusion:**

The methods of tattoo alignment and isocentre definition in cohort 3 reduced set‐up errors and improved isocentre reproducibility.

## Introduction

This three‐cohort quality improvement study aimed to decrease systematic and random set‐up errors and increase isocentre reproducibility by testing different methods of tattoo alignment and isocentre definition. The study developed as a result of the Sydney Cancer Centre's (SCC) participation in a quality assurance (QA)‐based Set‐up Accuracy Study (SUAS) developed by the Randomised Androgen Deprivation and Radiotherapy (RADAR) TROG 03.04 trial group.^1^ This process identified the need to assess and improve current practices to benefit patients as all departments have a duty of care to provide the optimal standard of care reasonably possible and that which meets international standards.^2^, ^3^ It is noteworthy that trial participation is a catalyst for assessing practices and implementing quality improvement studies. Haworth et al. estimated that 65% of centres participating in the RADAR SUAS made changes to daily practices to improve set‐up accuracy.^4^, ^5^

The pre‐study protocol at SCC for supine pelvis radiotherapy patients dictated that three tattoos be applied along a transverse plane at the computed tomography (CT) simulation. However, the alignment of these tattoos was not reproduced for daily treatment potentially introducing systematic set‐up error. The authors hypothesised this to be a major contributor to set‐up inaccuracy and speculated that the ideal patient set‐up be all tattoos aligned daily as per the CT simulation, with minimal manipulation of the patient's skin.^3^, ^6^ Further speculation detailed that a cranio‐caudal (CC) isocentre position as defined by the lateral tattoos would improve accuracy as compared to reliance on the anterior tattoo.

Information specific to tattoo alignment and isocentre localisation procedures for supine pelvis patients is scarce in the literature and although published “Best Practice Guidelines” outline acceptable planning and treatment methods, they are not prescriptive with respect to tattoo alignment and isocentre definition.^3^, ^7^ Hence, this study investigated the following: (1) Does aligning lateral tattoos, to minimise both roll and yaw, decrease set‐up error and (2) Does defining CC isocentre position at the lateral tattoos and left–right (LR) isocentre position at the anterior tattoo improve isocentre reproducibility?

## Methods

Between 2007 and 2009, three cohorts of patients were sequentially recruited. Cohorts 1, 2, and 3 recruited 11, 11 and 10 patients respectively. The study population consisted of patients with low or intermediate risk prostate cancer receiving radical external beam radiotherapy (EBRT). Study eligibility criteria stated that patients must complete at least 20 EBRT fractions. Patients with fiducial markers were ineligible as markers could introduce bias in image matching. Approval was granted by both the hospital's Ethics Review Committee and Trans Tasman Radiation Oncology Group (TROG). All patients gave their informed consent prior to participating in the study. RADAR guidelines utilised the van Herk method for calculating random and systematic set‐up errors and we followed this methodology.^8^, ^9^

### CT simulation and planning

**Figure 1:**
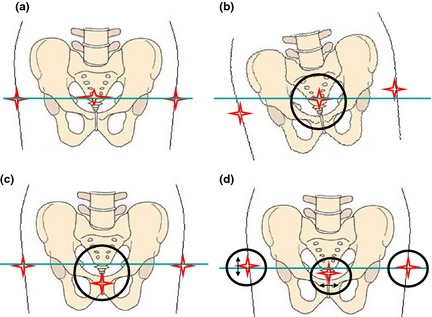
Demonstrates the tattoo alignment of all cohorts and illustrates pitch and yaw. The crosses depict tattoo locations and the circles represent the tattoos used for isocentre definition. (a) computed tomography (CT) simulation – Three set‐up tattoos administered in the same transverse plane. (b) Cohort 1 – Lateral tattoos are levelled to horizontal lasers only, to minimise roll. Note that this image demonstrates significant yaw. Isocentre is defined by the anterior–posterior tattoo. (c) Cohort 2 – Lateral tattoos are levelled to horizontal and vertical lasers to minimise roll and yaw. Note that this image demonstrates pelvic tilt i.e., pitch as the anterior–posterior tattoo is out of alignment with the laterals. Isocentre is defined by the anterior–posterior tattoo. (d) Cohort 3 – Lateral tattoos are levelled to horizontal and vertical lasers to minimise roll and yaw, and pitch is adjusted so that the anterior tattoo is within 5 mm of the lateral tattoos in the cranio‐caudal (CC) plane. Isocentre is defined by the lateral tattoos for the CC position and the anterior tattoo for the left–right position.

Patient position and immobilisation was standardised to address intra‐patient variability. Patients were instructed to have a comfortably full bladder and to have emptied their rectum prior to CT simulation. Patients lay supine on a flat carbon fibre couch top (CFCT). A Med‐Tec^™^ (CIVCO, Coralville, Iowa) kneeblock and footrest was indexed to the table top according to the patient's leg length and comfort. Straightening of the patient involved aligning the sagittal laser to the midline of the immobilisation devices, then shifting the patient laterally to match. Patient landmarks used included the xiphoid process, pubic symphysis and/or base of penis. Three set‐up tattoos were administered in the same transverse plane: the anterior tattoo was placed superior to the pubic symphysis and the lateral tattoos at the approximated mid‐pelvic separation (Fig. 1a). The entire pelvis was scanned with 3 mm image slice reconstruction.

### Treatment

Patient position, immobilisation, bladder and bowel preparation were as per CT simulation. Patients were prescribed up to 70 Gray EBRT at 2 Gray/fraction daily and treated with a five‐field 3D‐conformal radiotherapy technique executed on a Varian 21EX linear accelerator. Cohorts 1 and 2 were treated using a Mylar table top; a solid CFCT was used for cohort 3 due to changes in departmental practice. The methods of tattoo alignment and isocentre definition differed as follows:

- Cohort 1 patient set‐up aimed to minimise roll by aligning each lateral tattoo to horizontal lasers (without correction in the CC direction) (Fig. 1b). Radiation therapists (RTs) recorded the superior/inferior distance of each lateral tattoo from the anterior tattoo (after the patient was aligned in the treatment position) to later assess yaw (Fig. 3). The isocentre was defined by the anterior tattoo. At the first treatment, an anterior–posterior source to skin distance (AP SSD) was set to isocentre to gain a digital table height readout which was recorded and used for isocentre depth for the remaining treatments unless otherwise indicated by electronic portal images (EPI) results.
- Cohort 2 patient set‐up aimed to minimise yaw and roll by aligning lateral tattoos to each other using both the horizontal and vertical lasers (Fig. 1c). Isocentre alignment was as per cohort 1.
- Cohort 3 patient set‐up aimed to minimise pitch, yaw and roll by aligning lateral tattoos as per cohort 2, but also ensuring that the anterior tattoo was within 5 mm superior/inferior of the lateral tattoo plane by adjusting the patient's pelvic tilt (Fig. 1d). Isocentre was defined by lateral tattoos for CC direction and anterior tattoo for LR direction. Isocentre depth method was as per previous cohorts.

### Imaging

For each participant, 20 orthogonal pairs of EPIs (AP and left lateral) were acquired.^4^ EPIs were taken fraction 1 through 10, then alternate fractions. Imaging dose was accounted for to eliminate increased radiation exposure to patients. EPIs were manually matched offline to digitally reconstructed radiographs (DRRs) using bony anatomy references. One of two designated RTs performed the image matching and recorded data into spreadsheets later verified by physicists.

## Results

A total of 1280 EPIs were analysed. Two patients in cohort 1 were excluded as the treating radiation oncologist declared the patients non‐compliant, due to their inability to maintain a stable position during each treatment fraction.

Yaw was substantially reduced in cohort 2 as compared to cohort 1 by the alignment of the lateral tattoos in the CC direction (Fig. 3). Cohort 2 set up errors remained similar to cohort 1 (Table 1) as did isocentre reproducibility results within ±10 mm. However, cohort 2 isocentre reproducibility within ±5 mm decreased from 86.7% in cohort 1 to 79.2% (Table 2).

**Table 1:** Shows set‐up errors for all cohorts, including the predicted results for cohort 2 if isocentre was defined by the lateral tattoos for the CC position.

Mean Setup Error	Systematic (mm)			Random (mm)			Combined (mm)		
	LR	AP	CC	LR	AP	CC	LR	AP	CC
Cohort 1	0.9	−1.0	−1.3	1.8	2.5	3.1	2.4	3.1	3.4
Cohort 2	−0.1	1.0	−1.4	2.8	3.0	3.3	3.1	4.7	3.5
Cohort 2 (lateral tattoo prediction)	−0.1	1.0	−0.3	2.8	3.0	2.5	3.2	4.7	2.9
Cohort 3	0.0	−1.1	−0.5	1.7	2.4	1.4	2.2	3.1	1.8

LR, left–right; AP, anterior–posterior; CC, cranio‐caudal; mm, millimetres.

**Table 2:** Isocentre reproducibility was evaluated as per the RADAR SUAS criteria by which a percentage of treatment isocentres coinciding with planned isocentres within 10 mm and 5 mm was calculated.

Treatment isocentre coinciding with planning isocentre	±10 mm	±5 mm
Cohort 1	99.3%	86.7%
Cohort 2	98.5%	79.2%
Cohort 3	100.0%	92.1%

RADAR SUAS, randomised androgen deprivation and radiotherapy set‐up accuracy study.

Cohort 3 results were superior and met RADAR SUAS recommendations for set‐up accuracy and isocentre reproducibility. One patient in cohort 3 showed repeated pitch as compared to three patients in cohort 2. The set‐up errors reduced to <2.5 mm (1 standard deviation [SD]) for all directions (Table 1) and treatment isocentres within ±10 mm and ±5 mm of planned isocentre increased from 99.3% and 86.7% for cohort 1 to 100.0% and 92.1% for cohort 3 (Table 2).

## Discussion

**Figure 2:**
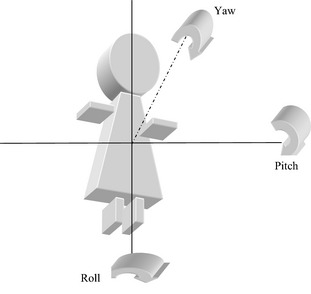
Illustrates pitch, yaw and roll as rotation about three axes.

Globally, many centres practicing daily image‐guided radiotherapy (IGRT) are limited to correcting translational errors via couch movement in three dimensions only. This inability to correct pitch, yaw and roll (Fig. 2) via couch movement means accurate in‐room patient tattoo alignment and isocentre definition remains valid and valuable in the IGRT era.^10^ Treatment units with six degrees of freedom are commercially available but access to all patients in still limited. Furthermore, tattoo alignment continues to play an important role in identifying correct treatment sites, in patient groups without fiducial markers, in cases where daily IGRT is not appropriate or necessary, and where daily IGRT is not available such as radiotherapy centres in developing nations.^11^, ^12^

This study provides evidence that simple changes can be implemented to improve treatment accuracy. Cohort 1 represented the set‐up accuracy of the departmental prostate protocol at that time. Cohort 1 systematic, random and combined CC set‐up errors of −1.3 mm, 3.1 mm, and 3.4 mm (1 SD) respectively are comparable to data presented by other groups who used the anterior tattoo to define CC isocentre position. Griffiths et al. analysed set‐up practices of 15 centres as a part of the MRC RT01 trial and reported that only 1/15 centres used the anterior tattoo for CC isocentre positioning and this centre showed the highest error amongst the group of 3.1 mm (1 SD) total error.^7^

**Figure 3:**
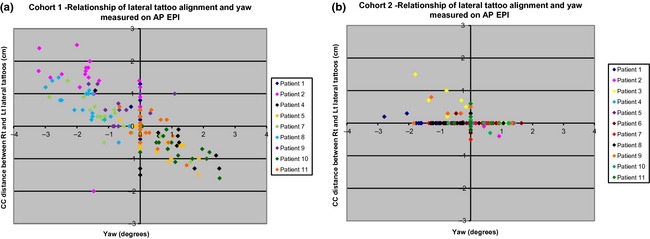
(a) Lateral tattoos were not aligned in the cranio‐caudal (CC) direction in cohort 1 patients. This shows that the greater the distance between the lateral tattoos in a CC direction, the greater the yaw as measured on AP EPIs. (b) Lateral tattoos were aligned in the CCdirection for cohort 2 patients resulting in reduced yaw measured on anterior–posterior (AP) electronic portal images (EPI). Rt, right; Lt, left

Assessment of yaw in cohort 1 showed that the greater the distance between the two lateral tattoos about the anterior tattoo, the greater the yaw shown on the AP EPI (Fig. 3a). Hurkmans et al. reported that reducing yaw is important as rotational errors greater than 3° can result in deformation of projected anatomy and therefore translational errors, thus impacting on image analysis and isocentre reproducibility.^13^ In cohort 2, the yaw measured on AP EPIs was greatly reduced due to the aligning of lateral tattoos (Fig. 3b). However, cohort 2 CC set‐up errors did not decrease nor did isocentre reproducibility increase upon cohort 1 (Tables 1 and 2). This strongly suggested that the major contributor to this error was setting CC isocentre position relative to the anterior tattoo which is subject to movement due to weight changes and daily bladder volume variations.^6^, ^13^, ^14^ Williamson hypothesised that aligning the lateral tattoos to each other and using these to set CC isocentre position would be more accurate as the “lateral tattoos represented two points in line with the approximate position of the isocentre and as such were less subject to movement than the anterior skin surface” (Fig. 1).^14^ Greer et al. focussed his study on isocentre depth accuracy but also noted that lateral tattoos were aligned to each other and defined CC isocentre position.^15^, ^16^ A correlation between the measured distance of the AP and lateral tattoos in the CC plane and the averaged CC isocentre position on paired AP and lateral EPIs of the same treatment fraction in cohort 2 patients supported Williamson's proposal. Cohort 2 data was then used to predict a reduction in CC set‐up error if aligned lateral tattoos were used to set CC isocentre position (Table 1). Griffiths et al. reported that 14/15 UK centres aligned lateral tattoos to minimise roll and yaw, with seven of those centres also aligning the anterior tattoo to the lateral tattoos to minimise pitch. 12/15 centres used the lateral tattoos for CC isocentre definition and reported an SD range of 0.9–1.8 mm set‐up error.^7^ This collective evidence prompted us to set the isocentre to the lateral tattoos for cohort 3.

Cohort 3 tattoo alignment and isocentre definition methods are in line with the United Kingdom (UK) as well as Australia and New Zealand (NZ) best practice documents.^3^, ^7^ For cohort 3 patients, lateral tattoos were aligned to minimise roll and yaw and pitch was also minimised by ensuring the anterior tattoo was within 5 mm of lateral tattoos in the CC direction. A 5 mm tolerance was considered acceptable as difficulty is often experienced with aligning the anterior tattoo to the lateral tattoos due to anterior tattoo movement vulnerability as previously discussed.^6^, ^14^ Cohort 3 results achieved the most noteworthy improvements in set‐up accuracy. Comparing cohort 2 to cohort 3 results revealed a reduction in CC systematic error −1.4 to −0.5 mm and random errors CC 3.3 to 1.4 mm and LR 2.8 to 1.7 mm (Table 1). The Hurkmans et al. review of clinical set‐up error data reported a SD range of 1.0–3.8 mm for prostate set‐ups with the CC and AP directions showing the greatest variance. Hurkmans et al. concluded that 2.5 mm (1 SD) for both random and systematic error is the “state of the art” accuracy which is achievable with or without immobilisation, using standard radiotherapy equipment and without daily set‐up accuracy corrections.^13^ Cohort 3 results meet this level of accuracy. Booth and Zavgorodni further tighten the target accuracy stating that a systematic error of 2.0 mm (1 SD) for prostate set‐up is achievable with random set‐up error being the limiting factor for tighter margins and more conformal treatment.^17^ Striving to further improve AP set‐up accuracy is of particular importance in the prostate setting to further reduce the incidence of rectal toxicity.^2^

Pitch was indicated by discrepancies in the CC axis values of 3 mm or greater between the AP and lateral EPI of the same patient for the same treatment fraction. (Due to changes to the offline image review software after cohort 2 data assessment, in‐plane image rotations i.e., pitch and yaw were no longer measurable in degrees of rotation). The EPIs of 1 patient in cohort 1, three patients in cohort 2, and one patient in cohort 3 were identified as repeatedly displaying pitch differing from the planning CT data set. This may account for the poor set‐up accuracy results in cohort 2 as pitch shifts the anterior tattoo (used for isocentre definition in cohorts 1 and 2) in the CC direction and creates greater uncertainty when matching EPIs to bony anatomy.^2^

The in‐house image verification protocol negatively impacted on set‐up error and isocentre reproducibility results. Orthogonal EPIs of the first three fractions were acquired and manually matched offline to DRRs to assess systematic errors. Isocentre displacements greater than 5 mm in any direction were averaged and applied as an isocentre correction prior to the fourth fraction meaning displacements up to 10 mm were not corrected for until fraction four. A resulting recommendation from this study to benefit future patients specified that displacement equal to 5 mm or more in any direction should be assessed and corrected pre‐treatment for fractions 1–3 and for weekly images.

All images analysed for set‐up accuracy were manually matched by one of two RTs only which may be considered a design weakness. van Lin et al. reported two RTs manually matched all images and inter‐observer differences were accounted for by setting an action level for image reassessment: greater than 5 mm in the CC direction on a lateral image or greater than 3 mm in any other direction.^2^ In this study, the image analyst reassessed images when discrepancies greater than 3 mm in the CC direction were recorded between the AP and lateral EPIs of the same treatment fraction.

Another study weakness is that a CFCT was implemented in cohort 3 due to departmental changes in practice at that time. This was anticipated to show a reduction in AP systematic error as all patients for all cohorts were simulated on a CFCT however, no such improvement was shown (Table 1).^13^ Setting AP SSD at the first treatment, as opposed to a measured height above table top (HATT) from CT simulation or the planning system, may have negated systematic error caused by using different couch surfaces.

In total, 34 isocentre corrections were applied across all cohorts. Here, 19/34 corrections were made in the CC direction during cohorts 1 and 2 when the isocentre was defined by the anterior tattoo. No CC corrections were required in cohort 3 when the isocentre was defined by lateral tattoos. Furthermore, in cohort 3, no LR isocentre corrections were required and only four AP corrections were required. The reduced number of EPIs showing 5 mm or greater displacements in cohort 3 proved this method of tattoo alignment and isocentre definition more accurate.

## Conclusion

These findings are relevant to all supine positioned pelvis patients treated on a treatment couch limited to translational correction capabilities in three degrees of freedom. Cohort 3 methods of tattoo alignment and isocentre definition decreased set‐up errors and increased isocentre reproducibility, hence improving our standard of care. Of note, these results also meet RADAR SUAS specifications and international standards of set‐up error less than 2.5 mm (1 SD).

The following recommendations for optimal tattoo alignment and isocentre reproducibility have been implemented into practice and protocol at SCC:

- Patients should have three reference tattoos – one anterior and two lateral tattoos in the same transverse plane
- To minimise yaw and roll, lateral tattoos should be aligned to each other in the CC and AP directions
- To minimise pitch, pelvic tilt should be adjusted so that the anterior tattoo is within 5 mm of the aligned lateral tattoos in the CC direction
- Lateral tattoos should define CC isocentre position
- Anterior tattoo should define LR isocentre position.

## Conflict of Interest

The authors declare no conflict of interest.
